# Supplementary material for: Are carbon emissions trading and green financial instruments synergistic? -Comprehensive quantitative research based on content analysis
Source: PLoS One. 2024 Mar 7;19(3):e0298601. doi: 10.1371/journal.pone.0298601 (PMC10919601; doi:10.1371/journal.pone.0298601)
Supplement: S1 Data — (ZIP) [file pone.0298601.s001.zip › Data/地级市Z_F工作报告.zip!/╔╜╬≈/┬└┴║╩╨/└┤╫╘╣1⁄2╓┌║┼í╛┬φ┐╦╩2╛▌═°í┐_2015.docx]

现在，我代表市人民政府向大会报告工作，请予审议，并请市政协委员和其他列席人员提出意见。

2014年工作回顾

过去一年，面对错综复杂的经济形势和艰巨繁重的改革发展稳定任务，我们深入学习贯彻党的十八大、十八届三中、四中全会和习近平总书记系列重要讲话精神，坚决落实省委、省政府各项决策部署，特别是王儒林书记对吕梁工作的重要指示，始终坚持市委的坚强领导，在市人大、市政协的监督支持下，团结和依靠全市人民，牢牢把握“打基础、利长远、惠民生”总体要求，沉着应对，逆势作为。全年完成地区生产总值1101.3亿元,同比下降2%；固定资产投资1017亿元，增长16.5%；社会消费品零售总额376.8亿元，增长9.5%；公共财政预算收入130.6亿元，下降20.35%；城镇居民人均可支配收入21485元、增长7.4%，农民人均可支配收入6754元、增长10%；居民消费价格上涨1.7%，城镇新增就业4.17万人，转移农村劳动力4.4万人，城镇登记失业率为2.54%。万元地区生产总值综合能耗下降2.28%；六项污染物减排和万元工业增加值用水量降幅年度任务全面完成。

一年来，我们主要做了以下工作：

——坚持把稳增长作为头等大事，全力以赴上项目、稳煤炭、保企业、增效益。

强化投资拉动。以项目投资拉动增长，深入开展“项目见效年”活动，“六位一体”统筹推进重点工程建设，项目签约、开工、建设、投产等指标均位居全省前列。全市288个省市重点工程完成投资1223亿元，其中省重点工程完成投资866.4亿元,全省排名第一。一批煤焦化项目循环发展，铝系项目快速崛起，装备制造项目稳步推进，电力项目多元发展，重大基础设施项目进展顺利，生态环保民生项目发展提速，发挥了稳增长的“火车头”作用。

稳定煤炭生产。全面清理规范涉煤收费，取缔涉煤收费15项，为企业减负4.29亿元。市级财政投入300万元培训煤炭企业职工，有效维护了管理层和技术队伍的稳定。加强煤炭监测调度，积极推行煤电联营，煤焦互保措施，市内电厂基本使用本地煤，达到560万吨；焦化企业80%的原煤来自本地，达到3200万吨。加强煤矿分类监管，推进矿井改造升级和达标建设，累计完成投资33亿元，竣工验收煤矿15座，新增产能2590万吨，实际生产1.16亿吨。煤炭开采及洗选业增加值达到396亿元，占全市工业增加值的57.1%,有力支撑了全市经济发展。

帮扶企业脱困。坚持市县两级领导包联重点企业,及时调度各类生产要素，多渠道帮扶企业发展。推进银企合作，协调金融机构为365户企业（项目）贷款160亿元。发挥财政资金的杠杆作用，中小微企业专项扶持资金由300万元增加到2000万元，减免税收1060万元，发放助保贷资金1.2亿元，扶持中小微项目138个，新创办中小微企业2267户，培育销售收入超亿元“小巨人”企业11户。中小微企业财税贡献占到全市公共财政预算收入的49%，成为市域经济增长的生力军。

——坚持把调结构贯穿经济工作始终，着力构建经济增长新格局。

加快传统产业改造升级。完成转型技术改造项目投资341亿元，建成试产或部分投产项目62个。淘汰水泥、炼铁、焦化等落后产能473万吨，停建违规项目9个、产能625万吨。加快焦化行业兼并重组，企业（项目）数减少28个，户均产能增加86万吨，孝义金州煤系针状焦项目竣工投产，一批产业延伸项目顺利推进。编制完成燃煤电厂规划，8个低热值煤电厂核准在建和取得“路条”，总装机容量达到540万千瓦，交城国锦2×30万千瓦1号机组并网发电。全市首座光伏发电项目在汾阳并网发电，3个风电项目在岚县、方山基本建成，全年发电量达到112.6亿千瓦时，增长13.3%；交口道尔、兴华等一批铝基新材料项目落地开工，铝系产品产量达到959万吨，增长33.6%，为我市建设电力大市和铝工业基地奠定了坚实基础。

加大新兴产业培育力度。完成新兴产业固定资产投资242.4亿元，以锂离子电池材料、太阳能备板膜和高铁动车制动盘等为标志的新材料与装备制造业提档增速。特别是在文水建成全省最大的太阳能电池及光伏组件项目，当年开工、当年投产，填补了我省在核心光伏发电装备制造领域的空白。以临县电商王小帮为代表的电子商务等物流新业态起步发展。旅游业发展进程加快,新增4A级景区5家，旅游总收入达到182亿元，增长27.7%。鼓励发展高新技术产业。出台扶持政策，减免高新技术企业税费1000万元。全市自行研究课题50项，引进消化吸收科技成果110项，煤沥青基等新技术研发与示范取得突破。新增有效发明专利31件，新培育高新技术企业7户，新增税收2亿元。引深与国防科技大学合作，“一院三基地”建设有序推进，科技研发取得新进展。

——坚持把改革开放作为强大动力，持续释放发展潜力。

转型综改试验区建设进展顺利，“1231”年度目标任务基本完成。特别是兴县肖家洼煤矿铁路专用线等30个重大项目全部开工，完成投资369亿元，11个建成投产。市县两级政府机构改革基本完成。深化行政审批制度改革，累计取消和下放审批事项28项，精简市级审批事项291项，压缩承诺时限近五分之四，单一事项审批时限控制到5个工作日。围绕重点领域和关键环节，初步建成统一、透明、规范的公共资源交易市场。加大招商引资力度，签约项目186个，到位资金713亿元。

——坚持把“三农”工作作为重中之重，努力促进强农惠农富农。

持续加大“三农”投入，全市农林水支出达28.5亿元。粮食总产量达12.6亿公斤，创历史新高。扎实推进“8+2”农业产业化和“一村一品”“一县一业”工程，种植面积增加25.8万亩，扩大2.8倍，带动25万农民人均增收2300元，初步建成9个特色产业基地县，建成234个专业村。实施精准扶贫，全市1439个贫困村、74万贫困人口建档立卡信息采集系统初步建成；推进产业扶贫，55个项目完成投资22.9亿元；开展机关定点扶贫和干部驻村帮扶工作。12.7万贫困人口脱贫，贫困发生率下降3.5个百分点。积极培育新型农业经营主体，新增市级以上专业合作示范社85家。完成生态林建设63.3万亩，成功举办全省造林绿化现场会。

——坚持把基础设施建设作为重要引擎，统筹推进城镇化进程。

大力加强基础设施建设。集中力量完成一批建设多年的重大工程。吕梁机场建成通航，旅客年吞吐量突破10万人次。太佳高速黄河大桥和西纵高速岢临、临离段建成通车，环城高速具备通车条件。瓦日铁路、太兴铁路通车试运行，吕临支线主体完工，除交口外，12个县（市、区）通了铁路。新改建农村公路304.7公里，新增通车里程175公里，全市农村公路通车里程突破1.5万公里。中部引黄工程及配套小水网、重点病险水库除险加固工程有序推进，龙门供水工程输水隧道全线贯通。解决15万人的饮水安全问题，完成水土流失治理45.4万亩。兴县500千伏和瓦日铁路配套的4个220千伏、3个110千伏输变电工程及6个牵引站建成投运。年内建设输气管网221公里，城市人口气化率达到77%。努力提高城镇化水平

努力提高城镇化水平。市区控制性详细规划编制完成并获得批复，交城等4县市总体规划和孝义、汾阳两市城镇体系规划以及全市城市燃气等12个专项规划编制完成。市区管理体制进一步理顺。主城区完成凤山公园循环路改造和滨河东路、呈祥路南延一期工程，城区学校附近新建4座人行天桥，极大地保障了学生的出行安全。新开工保障性安居工程20568套，建成20459套。新城区开工建设13个重点项目，吕梁大道一期工程11.47公里主线贯通，7座大桥基本建成，回迁安置楼和供水、供热、供气、火车站站前广场等基础配套工程稳步推进。新增城市绿化面积145.5公顷。岚县创建为省级园林县城，孝义创建为国家卫生城市。全市城镇化率提高1.56个百分点，达到44.67%。——坚持把民生事业放在优先发展位置，不断改善人民群众生活质量。

着力保基本、补短板、兜底线，全市民生投入达到221.65亿元，占到公共财政支出的84%。推进教育事业协调发展

推进教育事业协调发展。义务教育“全面改薄”工程启动实施，孝义通过国家义务教育均衡发展评估认定。全市高考二本以上达线1.23万人，创历史最好成绩。特殊教育学校建设迈出实质性步伐，县级职教中心建设扎实推进。落实助学资金5.93亿元，资助贫困学生9万人，占到学生总数的14%。支持医疗卫生事业发展

支持医疗卫生事业发展。交城等8个县级公立医院改革试点全面铺开。新农合筹资标准稳步提高，补偿参合农民10.7亿元。城乡居民大病保险制度逐步完善，补助大额门诊特殊病种增加5种，重大疾病医保病种增加4种。人均基本公共卫生服务经费财政补助标准提高到35元。重大传染病及突发公共卫生事件防控处置有力。促进人口均衡发展，269户家庭享受到“单独两孩”政策。

深入实施省市便民项目。改造农村危房2.1万户；培训新型职业农民4526人；新建城镇标准化幼儿园21所，改造农村幼儿园45所；易地扶贫搬迁工程入住1409人；清运农村垃圾129.5万吨，清理农村“四堆”1.9万处。我市自主实施的“便民六件实事”顺利收官。

全面加强社会保障。城镇职工和城乡居民养老、生育、工伤、失业、医保覆盖率均达到96%以上。农村五保户集中供养、分散供养标准每人每年分别提高1900元和500元，城乡低保保障标准每人每月分别提高25元和22元。全市农村低保对象达到30.52万人，基本实现了动态管理下的应保尽保。康复救助贫困残疾人5592名。为117.8万低收入农户供煤119.5万吨。启动实施“聚焦民生、服务群众”主题实践活动，初步解决民生诉求4.3万件。

积极发展文化事业和文化产业。文化惠民工程扎实推进，公共文化设施不断完善，群众文化活动丰富多彩。非物质文化遗产传承保护成效明显，新增3个国家级、10个省级非物质文化遗产项目。实施文化“走出去”战略，《廉吏于成龙》、《山里娃》等一批艺术精品广泛传播，文化产业发展水平进一步提升。

——坚持把安全作为发展底线，全力维护和谐稳定良好局面。

健全安全生产责任体系，严格执行重点行业安全生产领导挂牌责任制度，全面开展安全生产大检查、重点行业领域专项治理和“六打六治”打非治违活动，强化食品药品安全监管，全年未发生重大以上安全生产事故，危化品、特种设备等行业领域保持零事故。严厉打击和处置非法集资，有效化解金融风险。积极推进社会管理综合服务平台建设，深入开展治安突出问题专项整治，严厉打击各类违法犯罪活动，保持了社会和谐稳定。

——坚持把民主法制建设作为重要保障，加强和改进政府各项工作。

严格落实党风廉政建设责任制，持续加大反腐败斗争力度，严肃查处政府工作人员违纪违规和贪腐行为，以净化政治生态的实际成效取信于民。深入开展党的群众路线教育实践活动和学习讨论落实活动，认真践行“三严三实”，严格执行“八项规定”，坚决反对“四风”。开展“六权治本”试点工作，启动政府工作部门权力清单和责任清单编制工作。推进依法行政，建立每周例会制度，完善议事决策程序，政府工作的规范性进一步提高。自觉接受市人大的法律监督、工作监督和市政协的民主监督，全年办理人大代表建议156件、政协提案410件，办结率均为100％。加强审计监督，全市“三公”经费下降35.72%。支持各民主党派、工商联、无党派人士参政议政，工会、团委、妇联等人民团体作用得到发挥。

与此同时，粮食供销、外事侨务、文物保护、防震减灾、民族宗教、档案方志、应急管理、对台、气象等工作取得了新成绩。国防动员、人民防空、民兵预备役建设进一步加强。

各位代表，过去不平凡的一年，我们同舟共济，砥砺前行，成绩来之不易，奋斗蕴含艰辛，凝聚了全市人民的心血和汗水，更加坚定了我们奋发有为的决心和信心。在此，我代表市人民政府，向全市人民，向驻吕解放军、武警官兵、公安民警和驻市单位，向各民主党派、各人民团体和各界人士，向关心、支持和参与吕梁经济建设和社会发展的朋友们，表示衷心的感谢和崇高的敬意!

去年，我们遇到的困难比预期要大、要多，一些主要经济指标出现多年未有的负增长。客观上有宏观经济下行、金融环境趋紧等不利因素，根本上是我市多年积累的深层次矛盾和问题的集中暴露，同时还直接反映出我们各级政府在新常态下，战略思维滞后、信心勇气不足、应对能力不强。当前，我市“一煤独大”的畸重型产业结构还没有改变，传统产业产能过剩，转型升级步伐亟待加快；农村贫困面大、贫困程度深，农民增收渠道不多且极不稳定；基本公共服务水平仍然较低，民生事业历史欠账较多；财政收支矛盾进一步加剧，社会保障和政府融资偿债压力不断加大；维护社会和谐稳定、加强和创新社会管理任务艰巨；一些部门和行业“四风”问题尚未根绝，有的领导干部对工作消极敷衍、等待观望、畏首畏尾，部分工作人员不作为乱作为、懒政怠政、失职渎职依然存在，个别领域腐败问题还有发生。破解这些难题，保持经济社会平稳健康发展，是历史对我们的考验，更是人民对我们的期望。我们必须正视现实、勇于担当，夙夜在公、攻坚克难，不辱历史使命，不负人民重托！

2015年工作安排

今年是全面深化改革的关键之年，是全面推进依法治市的启动之年，是全面完成“十二五”规划的收官之年，也是我市全面实施“六权治本”、推进“六大发展”的开局之年，更是“深入学习贯彻习近平总书记系列重要讲话精神，净化政治生态，实现弊革风清，重塑吕梁形象，促进富民强市”十分重要的一年。

当前，挑战与机遇并存，压力与动力同在。中央基于“三期叠加”的阶段性特征，作出了我国经济发展进入新常态的重大战略部署。吕梁作为典型的资源型地区，去年以来，煤焦市场持续疲软，企业利润大幅下滑、资金周转压力加剧、生产经营异常困难，经济社会潜在的矛盾和问题日益凸显，并相互交织，目前仍在不断发酵、扩散。从国内外资源性产品市场释放的信号来看，我们今年面临的形势可能比去年还要复杂、承受的压力比去年还要大、遇到的困难比去年还要多。但是，我们更要看到稳定向好的一面。从宏观上看，中央和省稳增长、调结构的各项政策取向利好，全面深化改革的乘数效应和改革红利正逐步释放，新一轮对外开放、创新驱动和重点领域投资等后发优势将带来更多机遇。从我市来看，转型升级潜力巨大，资源型企业的市场地位正在转换，为新兴产业发展提供了空间，必将催生新的经济结构。发展理念发生转变，随着煤焦市场量价关系的理性回归和对经济新常态的深刻认识，重转型、调结构，向集约、质量要效益，已成为一大批企业家的共识。发展条件日趋完善，机场、铁路、高速公路等立体交通网络已经形成，电网、燃气、水利等基础设施建设步伐加快，发展瓶颈逐步打破。政府服务更加高效，高压反腐和“六权治本”的深入推进，使广大干部职工的灵魂受到震撼和荡涤，规矩意识正在成为政治自觉，政务环境进一步得以净化，新的发展秩序逐步形成。

正视新常态，抓住新机遇，引领新发展，对我们提出了全新的挑战和要求。我们必须坚定信心稳增长，不气馁，不灰心，不放弃，不动摇，保持战略定力和韧性，捕捉发展信号，多元增加动力，稳定经济增长；我们必须沉下心来打基础，以功成不必在我、甘于栽树的情怀和理念，担当奉献，摒弃浮躁，执着坚守，夯实基础，积蓄能量；我们必须蹄疾步稳促改革，简政放权，用政府权力的“减法”，换取市场活力的“乘法”，调动一切积极因素，创新驱动，让发展活力竞相迸发，让一切创造财富的源泉充分涌流；我们必须团结一致向前看，以战略的眼光、宽广的胸怀和开放的姿态，凝神聚力，逆势而上，积极探索转型路径、培育增长优势、谋求健康发展。

今年政府工作的总体要求是：认真贯彻党的十八大、十八届三中、四中全会和省委十届六次全会以及市委三届六次全会精神，以邓小平理论、“三个代表”重要思想、科学发展观为指导，以习近平总书记系列重要讲话精神为根本指针，按照“四个全面”战略布局，主动适应经济发展新常态，坚持稳中求进工作总基调，牢牢把握“打基础、利长远、惠民生”总体要求，以提高经济发展质量和效益为中心，在遏制下行态势、稳定经济增长上下功夫，在突出创新驱动、转变发展方式上下功夫，在全面深化改革、扩大对外开放上下功夫，在狠抓扶贫攻坚、保障改善民生上下功夫，在加强环境保护、推进生态文明上下功夫，加快实施“六权治本”，全力推进“六大发展”，实现吕梁经济平稳健康发展，社会和谐稳定。

综合考虑煤焦价格持续走低、财政非即期收入大幅减少等多方面因素，今年经济社会发展主要预期目标是：地区生产总值增长4％，固定资产投资增长16%，社会消费品零售总额增长10％，公共财政预算收入下降22％左右，城镇和农村居民人均可支配收入分别增长8%和9％，城镇新增就业岗位4万个，城镇登记失业率控制在4.2%以内，居民消费价格涨幅控制在3%左右。确保节能减排等约束性指标完成省下任务。

今年，我们要重点抓好以下十方面工作：

一、	千方百计应对严峻形势，努力遏制经济下行

竭力保障企业生产竭力保障企业生产。严格落实省政府减轻企业负担“60条”和我市相关政策，年内减轻企业负担23.3亿元。把解决企业困难作为政府工作的重点，坚持市县领导包联重点项目、重点企业，主动联系上级部门、组织经济部门、协调金融部门，完善监测预警机制，科学调度生产要素，为企业发展搭上一把手、添上一把柴、加上一把火。引导企业优化内部管理，千方百计降耗增效；创新营销策略，缩短资金回笼周期；加强同行业联合、上下游衔接，通过企业股权置换、采矿权转让、资本整合等多种方式，积极寻求战略合作伙伴，保障企业健康平稳发展；开展质量兴企，实施品牌战略，努力提升企业竞争力。

全力保持投资增长。多渠道增加全社会固定资产投资，持续增强经济发展后劲。围绕重大基础设施、产业转型、城镇化和生态环保、民生社会事业建设，突出抓好10大领域642个工程项目，确保年内完成固定资产投资1180亿元。充分发挥重点工程的支撑作用，深入开展“项目提质增效年”活动，完善项目推进机制，集中力量争取一批、招商一批、签约一批、开工一批重大项目，全力加快296个省市重点工程建设。同时，围绕强化弥补我市产业短板，加快培育新兴产业项目，引导资源型企业转型发展非煤产业和高新技术产业，不断优化投资结构。

深入实施煤炭革命。认真落实煤炭管理体制改革意见，推动煤炭产业向“六型”转变。加强市场预警，引导煤炭企业科学规避市场风险。实施科技兴煤，加快标准化、现代化矿井建设，推动临县霍州煤电、兴县锦兴等大矿完善审批手续，离石王家庄等15对矿井建成投产，控制高成本低质煤矿井生产，新增集约高效矿井产能1800万吨；加强与国内大型煤炭企业合作，积极参与煤炭期货和电子化交易，保障完善现货交易。加快煤炭产业延伸发展、煤化工链条式发展、煤炭固废综合循环利用，推进煤基产业多元发展。加强行业监管，实行煤电联营、煤焦互保，促进煤炭就地转化。依法依规推进联盛集团破产重整，帮助一批煤炭企业走出困境。

多措并举促进消费。实施养老家政、文化、旅游、住房等重点领域消费工程，激发和挖掘消费潜力，加快培育消费增长点。以铁路战略装车点和高速公路为轴线，合理布局相关产业，建设功能完备、流通畅达的物流基地。组织企业广泛参加“山西品牌中华行”等促消费活动，鼓励企业多渠道、多元化拓展产品市场。加大商贸流通企业培育、批发市场改造和乡镇集贸市场建设力度，优化城市快销物流仓储设施布局，积极发展农村现代物流。依托我市干鲜果、小杂粮、白酒等特色产品，推进电子商务平台建设，加快培育省级电子商务示范企业，打造一批特色电商村。深入开展规范市场秩序活动，净化市场环境，提振消费信心，努力增加本地消费指数。

面对前所未有的困难与挑战，我们不为任何风险所惧，不被任何干扰所惑，以坚韧不拔、自强不息的精神，切实做到非常时期尽非常之责、用超常之功创非凡之为，全力冲破经济下行困局！

二、坚定不移加快转方式调结构，促进产业优化升级

推进传统产业升级。一是坚持煤电一体化发展。推进交城等3个低热值煤电厂上半年并网发电；力争临县霍州煤电、中阳晋能桃园等6个电厂早日核准；加快兴县等4个2×100万千瓦坑口电厂前期工作，稳步推进电力外送工程建设。二是壮大新型煤化工产业。力争交城润景1.35亿立方米焦炉煤气综合利用、孝义金岩2×30万吨焦炉煤气制甲醇项目竣工投产，加快焦炭副产品由初级生产向精深加工转变。三是发展新型铝工业。加快建设交口、孝义、兴县三个铝业集中加工区，力争交口道尔200万吨铝系、兴华35万吨铝基新材料项目竣工投产，完成孝义、交口两个50万吨铝系项目前期工作，着力延伸铝土矿—氧化铝—电解铝—铝加工产业链条。四是实施汾酒辐射带动战略。推动杏花村酒业集中发展区建设，力争年内具备10万吨生产能力。同时，以杏花村汾酒引领区域白酒品牌，不断强化杏花村、汾阳乃至吕梁白酒的区域化概念，有效巩固和扩展白酒市场。

培育壮大新兴产业。装备制造业方面，推进孝义煤机制造、文水铸管、交城制动盘等项目投产达效，加快离石同辉重工5000台高射塑成型机系列、岚县继亨30万吨铸造技改项目前期工作。新材料、新能源产业方面，争取岚县、方山3个5万千瓦风电、孝义光伏发电项目并网发电；文水太阳能电池及组件二期和汾阳太阳能背板膜项目投产达效；新开工中阳等县光伏、风力和生物质能发电项目。做好国电吕梁市区垃圾发电项目前期工作。旅游业方面，加快方山北武当山、临县碛口、离石白马仙洞、中阳柏洼山等旅游景区标准化建设。高新技术产业方面，继续引深与国防科技大学合作，加快军民融合协同创新研究院科研成果转化，力争实现市场化交易。积极探索“互联网+”行动计划，促进云计算、大数据、物联网等与传统产业、现代制造业和现代农业相结合，努力提升产业化水平。

加快建设现代工业园。坚持新上项目进园区，推动企业集聚、产业集群发展。以交城、文水、孝义3个省级经济开发区为龙头，带动全市园区实现企业集聚循环、产业集群发展。进一步健全完善园区管理体制和工作机制。科学确定园区主导产业，实行差别化发展，形成合理的资源配置和产业分工格局。支持园区开放式发展，选择性承接东部转移产业，吸引具有自主创新能力和核心竞争力的企业入驻园区，加快提升各类园区产业素质。

畸重型产业结构和粗放型增长方式给我们带来深刻教训，我们就是要变“危”为“机”，抓住产业格局调整契机，利用市场倒逼机制，加快构建现代产业体系，用今天的结构大转型打造明天的竞争新优势。

三、全面深化改革扩大开放，不断激发发展动力和创造活力

加快转型综改试验区建设。实施“5121”年度行动计划，抓好以工商资本下乡为重点的5项重大改革，“四基三带一核心”为重点的10项重大事项，天源物流中心为重点的20个重大项目和吕梁工业经济发展趋势研究重大课题，靶向聚焦、精准施策，全面完成三年综改方案提出的各项任务。

深化重点领域改革。深化煤炭等产权交易制度和要素市场改革。加快矿业权管理等重点领域和资源配置等关键环节制度建设，保障公共资源交易平台健康运行。加快中小微企业综合配套改革。支持中小微企业创业基地建设，鼓励企业以技术和管理双轮驱动增加效益、提升素质。力争新创办1500户中小微企业，新增30户规模企业、15户“小巨人”企业。同时，突出抓好民营企业家和现代管理团队建设，引导民企建立现代企业制度。深化财税管理体制改革。严格执行新《预算法》，坚持先有预算、后有支出，推进预决算公开。扩大营改增范围，完善煤炭资源税从价计征县市分享体制。健全财政专项资金管理办法，加强政府债务管理。推进投融资体制改革。加强政银企合作，引导各金融机构创新产品，支持地方经济发展，确保信贷增量高于上年。推广政府和社会资本合作 PPP 模式，鼓励 BOT 模式。积极引进各类金融机构，规范发展农村商业银行、小额贷款公司，加快构建新型地方金融体系。推进社会信用体系建设，积极防控和化解金融风险。深化行政体制改革。坚持简政放权、放管结合、优化服务“三管齐下”，继续取消、下放、调整行政审批事项，全面清理非行政许可审批事项，坚决打通政策落实的“最先和最后一公里”。深化商事制度改革，推进司法、行政执法、文化等管理体制改革，推动事业单位分类改革和人事制度改革，释放发展活力。

实施创新驱动战略。加大科技研发投入和扶持力度，年内培育科技创新型试点企业10户、高新技术企业10户。增强企业自主创新能力，力争在煤焦、冶炼、农产品加工和生物资源开发等领域取得突破。实施人才战略，支持院士、博士后工作站建设，培养和引进一批产业领军人、职业经理人、学科带头人和高技能人才，用高端智慧为转型发展助力导航。统筹推进各领域科技体制机制创新，营造公平、开放、透明的创新环境。

加快对外开放步伐。树立合作共赢、抱团取暖的理念，向开放要生存、求发展。主动融入“一带一路”，积极对接京津冀协同发展等战略，加强与先进发达地区交流合作。大力提升对外贸易水平，建设汾阳核桃、临县有机红枣等8个省级外贸转型升级示范基地。积极推行跨境电子商务，争取25户进出口企业入驻第三方电子商务平台。建立健全政府牵头、企业为主、各类组织广泛参与的招商机制，多形式开展招商活动，力争完成项目签约2900亿元。

四、毫不放松抓好“三农”工作，努力增加农民收入

夯实农业发展基础。严格落实各项强农惠农政策，坚守788万亩耕地红线，粮食总产量稳定在9.5亿公斤以上。做好粮食储备、轮换和购销管理工作，确保粮食安全。培训新型职业农民4000人。启动实施农业综合标准化示范市创建工程，重点发展无公害、绿色、有机和地理标志产品，提高农产品质量和安全水平。扎实推进小流域综合治理开发试点工程，实现“治一方水土，兴一方经济”目标。

深入推进“8+2”产业化工程。一是规模化发展八大产业。建设核桃标准化示范园6万亩、高接换优1.5万亩；红枣标准化示范园2万亩，重点支持红枣抗裂果研究和推广；新发展食用菌2000万棒、绿色杂粮7.1万亩、绿色马铃薯5万亩、中药材等林下经济12.5万亩；推广应用设施蔬菜新品种、新技术1.5万亩。改良黄牛2万头，引进湖羊5500只，开展畜牧配方饲料推广和分段分群养殖试验示范。二是打造特色产业基地。因地制宜，重点建设汾阳核桃、沿黄四县红枣、兴县杂粮、岚县马铃薯、文水梨果、交口食用菌、中阳肉羊和生猪等一批特色产业基地县。三是培育壮大龙头企业。鼓励资源型企业进入农产品加工领域，重点培育一批辐射带动能力强的龙头企业。加强品牌宣传，壮大营销队伍，鼓励农超对接，拓宽营销渠道，努力增加我市特色农产品的市场份额。

加大扶贫开发力度。我市农民收入不达城镇居民的三分之一，近一半的农村还是贫困村，扶贫攻坚一刻也不能懈怠。要坚持精准扶贫、精准脱贫，突出重点目标、人群、产业和区域，因地制宜、因户施策，不断增强脱贫针对性和实效性。继续加大产业扶贫力度，扎实推进金融扶贫和贫困劳动力就业培训，推广光伏扶贫、电商扶贫、旅游扶贫等模式，探索贫困群众稳定受益机制。加快易地扶贫搬迁进程，推进贫困地区社会事业发展，提高基本公共服务水平。落实领导干部包村增收和机关单位定点扶贫措施，鼓励社会力量参与扶贫开发，减少贫困人口10.6万。

深化农村综合改革。一是开展农村土地承包经营权确权登记颁证工作。力争年内确权登记颁证村数达到40%。加快推进农村集体建设用地、宅基地使用权确权发证工作，年内发证率达到80%以上。二是培育壮大农业经营主体。力争发展省市县三级合作示范社160家、示范家庭农场35个、种植大户100户。三是分类推进农村土地制度试点改革。制定完善缩小征地范围办法，建立土地增值收益合理分配制度，完善被征地农民保障机制，探索农民住房保障新机制。四是不断深化水利、林业、供销社等改革，加快推进以高风险社为重点的农村金融体制改革。

五、积极稳妥推进新型城镇化，大力提高城镇化质量

协调推进市区一体化建设。推进城镇化，核心是人，关键是提高质量。要坚持主城区、新城区一体化发展，统筹规划建设宜居城市。一是坚持总规统领。编制完善城市详细规划，坚持产业发展、土地利用、生态环境等多规合一，实现城市粗放扩张型发展向限定城市边界、优化空间结构转变。二是加快主城区提质。启动实施呈祥路、滨河东路南延二期工程和碧水路大桥、马茂庄大桥、人行天桥等一批重点市政工程。完成20项市政维护工程、22项园林绿化工程。三是完善新城区功能。积极推进东属巴小学、大武小学、大武中心医院等民生项目，加快建设水厂、供热、供气、道路等项目，年内供热、供气、供水等管线全部贯通，安置群众顺利回迁，吕梁大道上半年通车。力争全市城镇化率提高1.8个百分点，达到46.47%。

加快大县城、重点镇建设。重点抓好城乡规划、村镇建设工作。汾阳和孝义两市控规覆盖率达到50%，其它县达到40%，编制完成各县市住房保障、排水防涝、防灾减灾等专项规划。大力加强县城市政公用设施、公共服务设施、公园绿地、中心街市、居住社区建设，推进县城内涵式发展。加快汾阳杏花、孝义梧桐、临县碛口、交口双池等30个重点镇建设，努力提高重点集镇吸纳人口、集聚产业的综合承载能力。

着力改善城乡人居环境。一是深入推进农村人居环境改善“四大工程”。完成6.5万户广播电视卫星户户通工程；推进3.85万人的采煤沉陷区治理，完成1.7万户困难家庭危房改造，新启动743户地质灾害治理搬迁工作；清理县城周围20公里范围内村庄生活垃圾；创建49个美丽宜居示范村。二是启动实施城市人居环境改善“四大工程”。加快设施提升、城市安居、城中村改造和环境提升，改造市区城中村片区，开工建设12720套保障性住房，基本建成8200套。加大市政地下管网建设改造力度，集中实施城市供水、燃气、供热管网和道路改造工程，年内率先完成市区、孝义、汾阳地下管网普查、综合规划和信息平台建设。市区新增供热面积400万平方米，全市集中供热和燃气普及率达到80%，供水普及率达到93%。全市新增城市绿化面积140万平方米，建成区绿化覆盖率达到37%。三是扎实开展城乡环境整治百日攻坚专项活动。全面实施城区“五大战役”、农村“四项攻坚”，大打一场城乡环境整治攻坚战。积极探索现代城市管理模式，重点在完善体制机制和管理队伍建设上取得新突破，规范以物业管理等为重点的房地产市场秩序，努力提高城市宜居软实力。

六、全面推进基础设施建设，进一步增强发展保障能力

推进交通建设。铁路方面，确保瓦日铁路、太兴铁路、吕临支线正式通车，加快沿线战略装车点建设，上半年形成1800万吨以上运力。开通吕梁—太原城际动车，抓好孝义—交口—石楼铁路前期工作。公路方面，确保环城高速通车、新国道太克线临县苗家庄至梁家会段升级改造工程开工建设，力争离石—祁县高速公路开工建设。加快太佳高速临县、岚县连接线，国道307柳林县城段和省道340中阳段改线工程建设。新建改造农村公路700公里，加强乡村公路养护，引深文明路创建。开工建设离石下安一级汽车站，加快县级客运站建设。

推进水利建设。加快中部引黄工程和涉及9个县（市、区）的供水网络体系建设。力争柏叶口水库龙门供水工程具备通水条件，千年水库竣工验收；做好碛口水利枢纽工程前期工作，争取列入国家“十三五”规划。推进规划内24座小水库前期工作，加强农田水利、病险水库（闸）、中小河流治理和水保建设，完成水土流失治理40万亩。全年万元工业增加值用水量下降2.5%，压减地下水开采量600万立方米。推进电网建设

推进电网建设。。加快推进特高压电网和汾孝区500千伏输变电工程前期工作。完成离石吴城、石楼罗村等10个110千伏输变电建设和改造项目。全面投运瓦日铁路配套供电工程。投资3.9亿元改造城乡中低压电网，努力提升我市供电和电网外送能力。

推进气化建设。重点建设柳林、石楼等煤层气开发利用项目区。加快临县、兴县等5个煤层气液化工厂建设步伐，力争形成日加工200万立方米的规模。完成临临线石楼西连接线等4条煤层气输气管线收尾工作。年内全市煤层气产能达到14亿立方米，稳步提高气化人口覆盖率。七、尽心竭力保障和改善民生，推动社会和谐进步

促进教育全面协调发展。加快实施学前教育第二轮三年行动计划，建设城区标准化幼儿园21所，改造农村幼儿园39所。深入实施义务教育“全面改薄”工程，离石、交城、汾阳、交口年内通过国家义务教育均衡发展评估认定。完成县级职教中心建设。深化教育综合改革，严格落实义务教育“就近、划片、免试”入学招生政策，完善普通高中和中职招生制度，支持民办教育发展。倾斜发展农村教育，实施乡村教师素质提升计划，创建200所农村素质教育特色示范学校。加快各类学校内涵式发展，重点加强教师队伍建设，建立健全教师评估考核体系，全面推进教育信息化工程。

提升医疗卫生服务水平。恢复运行市中医院，启动运行120急救中心，开工建设市疾控中心，加快筹建吕梁护理职业学院。县级公立医院改革实现全覆盖。推动优质资源向基层倾斜，启动县乡医疗联合体试点工作，加强医疗服务能力建设，努力提升专业队伍素质。加快乡村和社区卫生服务机构标准化、规范化建设，开展分级诊疗，方便群众就近就医。加强疾病防控、妇幼保健和爱国卫生运动工作。扎实做好人口计生工作。积极开展适龄儿童接种疫苗和农村60岁以上老年人免费健康体检等公共卫生服务。深入推动食品药品安全专项整治，确保群众“舌尖上的安全”。

保持就业基本稳定。全面落实就业创业扶持政策，建立完善服务体系，重点做好高校毕业生、农村转移劳动力、城镇困难人员等群体的就业工作，切实维护就业人员的合法权益。加强政府投资、招商引资和重大项目新增本地就业岗位审核，加大政府购买公共服务岗位力度，引导企业积极承担社会责任，努力保岗位、保就业。鼓励和支持困难企业采取在岗培训等办法，稳定就业岗位。支持大众创业、万众创新，鼓励“草根”创业，在政策、融资等方面提供服务，让创业门槛更低、创业天地更宽。

扩大社会保障覆盖面。启动实施全民参保登记、机关事业单位养老保险和城乡居民大病保险制度改革。推行城镇基本医保和生育保险费用省内异地就医即时结算。进一步提高社保待遇水平，企业退休人员人均基本养老金提高10%；城乡居民基础养老金每人每月最低标准由65元提高到85元；城镇居民基本医保和新农合人均年财政补助标准由320元提高到380元。全面落实临时救助制度，继续提升城乡低保、农村五保、优抚对象等群体的救助水平。关爱农村“三留守”群体，加强贫困残疾人康复救助，加快社会化养老服务体系建设。

加快文化事业和文化产业发展。。培育和践行社会主义核心价值观，大力加强公民道德建设。保护和抢救本土文化资源，挖掘整理晋绥红色文化、于成龙廉政文化和民间传统文化，推进非物质文化遗产传承保护，精心组织“永远的晋绥”等纪念抗战胜利70周年系列活动。加快文化基础设施建设，推进市级群众艺术馆、图书馆和县级文化场馆、基层综合性文化服务站所建设。实施政府购买公共文化服务惠民工程，丰富群众文化活动，倡导全民阅读。鼓励和扶持文艺精品创作。加强文化市场监管和广电新闻出版版权管理，推动体育事业健康发展。加快文化产业与科技、旅游、信息、物流等相关产业融合发展。加大文化精品宣传推介力度，不断提升区域文化传播力，重塑吕梁新形象。

加强和创新社会治理。。扎实开展平安创建行动，创新社会治理方式，健全立体化社会治安防控体系，严密防范和依法严惩各类违法犯罪行为。深入开展普法教育，切实增强全民守法意识。落实重大决策社会稳定风险评估机制，防止侵害群众利益现象发生。畅通信访渠道，落实信访积案化解工作责任，依法妥善处理信访活动中的违法行为。充分发挥社区居委会在社会管理中的积极作用，严格落实新建住宅小区配建居委会办公场所等政策，努力实现网格管理全覆盖、自治服务无缝隙。积极防范和处置突发性群体事件。依法强化网络管理，全面加强应急管理和防灾减灾能力建设。

发展各项社会事业。重视国防动员、国防教育、人防和兵役工作。加强文物保护、抗震设防、民族宗教、档案方志工作。支持工会、团委和妇联等人民团体工作。做好统计、老龄、慈善、外事侨务、对台、气象等工作。

各位代表，为民办实事、解难事是政府最大的责任。今年，我们要继续深入开展“聚焦民生、服务群众”主题活动，着力解决好群众的切身利益问题，集中力量办好“十件民生实事”。一是加快农村污水处理步伐，开工建设50个行政村生活污水管网工程。二是推进农村养老服务事业发展，新建100个农村老年人日间照料中心。三是改善公共交通服务，推进市区公交运营体制改革。四是着力稳定乡村医生队伍，每个行政村卫生室补助2000元，实现农民小病不出村。五是启动城区学校（幼儿园）标准化厕所改造，完成59所学校（幼儿园）旱厕改水厕工程。六是保障城乡居民用水，彻底解决主城区供水不足问题和8.07万农村居民以及8100名农村学校师生的饮水安全问题。七是建立设施蔬菜安全监管机制，实现全市设施蔬菜检验监测全覆盖、准出产品全贴标。八是完善便民服务网络，建立市县乡三级便民服务平台，对群众诉求统一受理、集中办理。九是不断丰富群众文化生活，每县（市、区）新建一个标准化公共文体活动场所。十是加快推进农业保险，试点推广红枣保险50万亩，实现设施蔬菜和玉米保险全覆盖。民生工作无小事，服务群众永远在路上。我们要始终把群众冷暖忧乐放在心头、扛在肩上，保障和改善民生不减力、不降速，努力增进人民福祉，让广大群众得到实实在在的好处！

八、深入持久推进节能减排和环境治理，切实加强生态文明建设

强化节能降耗。坚决淘汰落后过剩产能，推进重点领域和行业节能降耗工作。发展节能环保产业，开展企业节能低碳行动，实施一批节能改造示范项目，加强新建项目能耗达标管理，严格执行节能评估和审查制度，努力实现企业节能降耗和提质增效的高度统一。

强化环境保护。国家实施“史上最严”《环保法》，对传统发展方式提出了全新挑战。我们要严格落实环境保护目标责任制，加强农业面源、工业和城市大气污染防治以及水环境治理。全面完成城镇生活污水收集管网建设攻坚行动任务，确保离石等5个污水处理厂和配套污水收集管网建设工程年内竣工投运，全市污水处理率达到81%。加强燃煤型营业性炉灶清洁能源改造和燃煤锅炉、黄标车、老旧车辆淘汰等工作。建立新建项目污染物排放总量置换制度，严格控制新增污染物排放量。深入开展环保大检查活动，加强环保执法监管，坚持“零容忍”、出重拳、用重典，严厉查处违法排污、破坏生态行为，让排污企业付出代价、不敢违法，努力让蓝天常在、青山常在、绿水常在。

强化生态文明建设。按照“三加强三不减”要求，持续实施造林绿化“六大工程”，重点完成生态林建设50万亩，完成环城高速绿化，推进临离、岢临和太佳高速绿化，提升国省道、县乡村道路和社区绿化覆盖率。健全生态保护补偿机制，实施生态脆弱地区修复工程，切实做好水源地保护工作。倡导资源节约、绿色低碳、文明健康的生活方式和消费模式，提高全社会生态文明意识。

绿水青山就是金山银山，我们必须用最硬的手段、最严的处罚,铁腕治理环境污染，让人民群众呼吸新鲜的空气，喝上干净的水，在良好的环境中生产生活！

九、高度重视安全生产，推动安全形势稳定好转

始终绷紧安全生产这根弦始终绷紧安全生产这根弦。越是经济形势严峻复杂、越是改革发展任务艰巨、越是安全形势好转，越要高度重视安全生产。要强化安全生产是最大的民生、是比泰山还重的责任、是任何人任何时候任何地方都不可逾越的红线理念，坚持“三个敬畏”基本要求和“三个决不能过高估计”基本判断，按照“党政同责、一岗双责、齐抓共管”和“管行业必须管安全、管业务必须管安全、管生产必须管安全”的要求，进一步完善安全生产责任体系，严格落实“一票否决制”，层层传递压力，推动政府安全监管和企业主体责任落实到位，实现安全责任无缝对接。

始终坚持安全工作不放松。全面深入开展安全生产大检查，强化源头治理，实现隐患闭合管理。深化煤矿、非煤矿山、道路交通、消防和危化品、人员密集场所以及特种设备等重点行业领域专项整治。全面加强安全生产“三基”建设，切实提高安全保障能力。始终保持打非治违高压态势，依法维护安全生产良好秩序。坚决杜绝重大事故，遏制较大事故，减少一般性事故，促进全市安全生产形势持续稳定好转。

十、深入推进“六权治本”，全面加强政府自身建设

推进依法行政。牢固树立法治思维，把政府工作全面纳入法治轨道，努力建设法治政府。要尊法学法守法用法，不断提高运用法治方式深化改革、推动发展、维护稳定的能力和水平。坚持法无授权不可为、法定职责必须为，编制市政府工作部门权力清单、责任清单，推进政府机构、职能、权限、程序、责任法定化，规范行政自由裁量权，坚决消除权力设租寻租空间。按照法定权限和程序履职用权，严格行政执法复议、考核、追责，以法律和制度管人管事管权。科学编制“十三五”规划，使规划成为凝聚各方力量和指引转型发展的项目库、路线图和任务书。

强化权力监督。主动接受人大、政协、司法、社会和舆论监督，努力建设阳光政府。加强重点领域、重要岗位、关键环节和重点人员监管，推动公共资金、国有资产资源和领导干部经济责任审计全覆盖。完善纠错问责机制，健全问责制度和程序。严格执行“三重一大”集体决策制，全面推行行政权力运行、财政资金、公共资源配置、公共服务和公共监管等各类信息公开，加强电子政务建设，方便群众办事和监督。

加强廉政建设。坚持从严治政，努力建设廉洁政府。严格落实政府系统党风廉政建设责任制和党委党组主体责任，严守政治纪律和政治规矩，把纪律挺在前面，做到心有所畏、言有所戒、行有所止。进一步巩固党的群众路线教育实践活动和学习讨论落实活动成果，认真执行中央“八项规定”，坚决防止“四风”变异反弹。切实增强过“紧日子”的思想，厉行节约，反对浪费。始终保持惩治腐败高压态势，重点查处领导机关和领导干部中官商勾结、权钱交易、权权交易等问题，严厉惩处损害群众利益不正之风，坚决查办政府部门及其工作人员懒政怠政等“软腐败”行为，切实做到干部清正、政府清廉、政治清明。

狠抓工作落实。坚持问题导向，靠前站位，积极作为，努力建设务实政府。扎实开展“三严三实”专题教育，把推动工作落实作为政府工作常态。强化效率意识，坚持高标准、严要求，以“人一之、我十之”的努力，为企业、基层和群众提供优质高效服务。强化责任意识，坚持忠于职守、勤勉尽责，对确定的目标任务，以钉钉子精神一抓到底，坚决向无所事事、敷衍塞责、渎职失责、得过且过、应付了事等不良现象开刀，以督查倒逼工作进度，以追责倒逼责任落实。强化公仆意识，恪守为民之责、力行为民之举，健全完善各级干部深入基层调查研究、服务群众等制度，实实在在把群众的事情办好。强化大局意识，既要强调班子团结，也要搞好部门之间的合作，切实增强政府工作的凝聚力和战斗力，形成抓落实的强大合力。

各位代表，面对新形势、新任务、新要求，我们深感责任重大、使命光荣。让我们在省委、省政府和市委的坚强领导下，凝心聚力、锐意奋进，坚决完成经济社会发展各项目标任务，为开创弊革风清、富民强市新局面而努力奋斗！
